# Supplementary figures and images for: Dissociable effects of attention vs working memory training on cognitive performance and everyday functioning following fronto-parietal strokes
Source: Neuropsychol Rehabil. 2018 Dec 20;30(6):1092–114. doi: 10.1080/09602011.2018.1554534 (PMC7266670; doi:10.1080/09602011.2018.1554534)

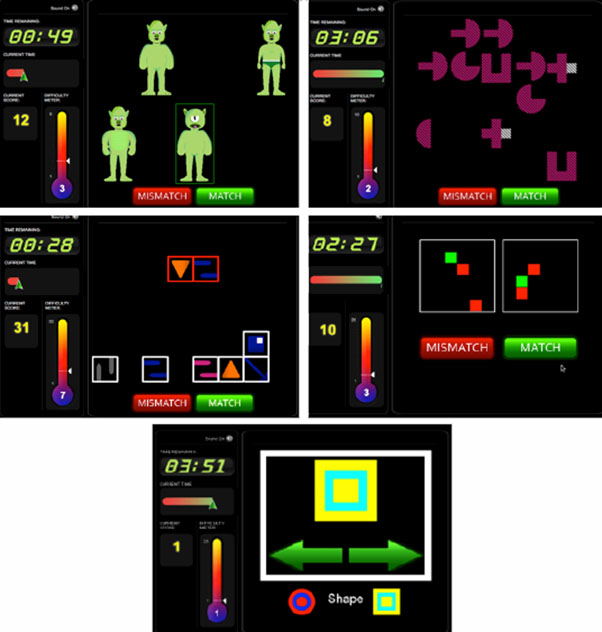

Supplement: Supplementary Materials [file PNRH_A_1554534_SM9365.jpg]
